# A political economy analysis protocol: Case study implementing nutrition and sustainability policy into government food procurement

**DOI:** 10.1371/journal.pone.0274246

**Published:** 2022-09-09

**Authors:** Maddie Heenan, Stephen Jan, Katherine Cullerton, Janani Shanthosh

**Affiliations:** 1 The George Institute for Global Health, University of New South Wales, Sydney, New South Wales, Australia; 2 The Australian Prevention Partnership Centre, Sydney, New South Wales, Australia; 3 Australian Human Rights Institute, University of New South Wales, Sydney, New South Wales, Australia; 4 School of Public Health, University of Queensland, Brisbane, Queensland, Australia; Universita degli Studi della Tuscia, ITALY

## Abstract

Most Australian state and territory governments have healthy food provisioning policies targeting availability of unhealthy food at the retail level, and sustainability policies promoting a life-cycle approach to procurement. However, it remains unclear if health and sustainability are important considerations in awarding contracts, and whether these high-level policies are implemented into supplier contracts. A political economy analysis framework has been developed to prospectively identify and explain barriers and enablers to policy implementation. Using food procurement in Queensland and South Australia as case studies, the political economy analysis seeks to understand the structural and contextual factors, bargaining processes, stakeholders, and incentives and ideas surrounding food procurement. It involves a desktop and content analysis of existing policies and food contracts, and key informant interviews with government and industry stakeholders. Participants will be targeted across different departments (e.g. health, environment, treasury) and in varying roles from policy design, contract management and food service, and industry suppliers in different food and drink categories (e.g. meat, packaged foods, beverages, fruit & vegetables). Participants will be recruited using purposive sampling. Thematic analysis of interview transcripts will be undertaken, informed by the political economy analysis framework. The study will identify current food procurement policy implementation barriers and enablers, including why high-level policies aren’t embedded into contracts, mechanisms for achieving policy coherence and future opportunities for addressing barriers and incorporating socio-economic, public health and environmental considerations into purchasing practices. Ultimately, the study will achieve impact by informing a whole of government approach to health and the environment by elevating the priority of health and sustainability in procurement (short term), increasing the availability of healthy and sustainable foods (medium term), and improving health and environmental outcomes (long term). To our knowledge this is the first political economy analysis of food procurement in Australia.

## Introduction

There is widespread agreement that current global food systems are unsustainable and that systemic change is needed to deliver equitable, healthy and sustainable diets [1, 2]. However, the complex nature of food systems makes policy change difficult with entry points at several levels of the system–the supply chain, food environment, individual factors and consumer behaviour [1]. Lack of political commitment or political will is frequently described as a barrier to policy change [3]. Achieving political commitment involves the presence of knowledge and evidence, politics and governance, and capacity and resources, and can range from rhetorical commitment to operational and ultimately embedded or system-wide commitment [4, 5].

Achieving system-wide commitment can be difficult when the competing interests of different government agencies are not aligned. This is described as policy incoherence. For instance, healthy food supply (a priority of the Department of Health) and sustainable business and consumption practices (a priority of the Department of Environment) are often seen to be at odds with economic growth (a priority of Treasury), thereby presenting incoherence within the government’s policy agenda [6, 7]. Aligning the objectives of health, sustainability and economic growth is paramount to achieving policy coherence, defined as ‘the systematic promotion of mutually reinforcing policies across government departments to create synergies towards achieving agreed objectives and to avoid or minimize negative spillovers in other policy areas’ [8].

Policy coherence and political will are interrelated and can be both barriers and enablers for change. Public health experts are more likely to achieve policy change and see their recommendations implemented by governments if they have a better understanding of the political processes and trade-offs involved [9]. These issues are context specific and therefore the changes that need to be made to address these potential barriers are likely to differ from setting to setting. Additionally, any reform is likely to create resistance from one or more interest groups. This includes industry interests; whose direct imperative is to turn a profit and not necessarily to engage in or promote a healthier food system. The objectives of commercial enterprises may be at direct odds with public health, and with certain government policies. This is what political economy analysis seeks to resolve.

Political economy analysis is a methodology used to identify and explain the barriers and enablers to policy reform and situate laws, policy or program interventions within an understanding of political, economic and social structures and processes. Most importantly a political economy analysis suggests opportunities to address barriers and mechanisms of incoherence to improve outcomes. A political economy analysis can be retrospective (explain policy decisions/ effects in the past) or prospective (analyse the current situation and propose strategies for action), and can be conducted at different stages of the policy cycle–agenda setting, policy design, policy adoption and implementation [10]. They seek to understand why the problem persists and what existing structures are relevant to the problem; what incentives and motivations shaped by these structures influence behaviour leading to the problem; and what actions can address the problem.

In assessing the policy problem, a political economy analysis is used to identify the structural and contextual factors, bargaining processes, stakeholders and incentives and ideas. It attempts to move beyond the ‘lack of political will’ problem to unpack the issue in greater detail [11]. This study is particularly concerned with the politics of implementation and uses government food procurement as a case study to understand the political economy surrounding healthy and sustainable sourcing of foods.

### Food procurement

The way in which food is sourced, purchased and provided by institutions or organisations is known as food procurement. It is the act of professionally managing the purchasing and acquisition of goods, services, works and utilities [12] and is the linking of supply chains and food environments. It is for this reason that food procurement policies offer an opportunity to address issues at higher levels of the food system and target the supply chain and food environments collectively. The United Nations Food and Agriculture Organisation (UN FAO) and the World Health Organization (WHO) recommend sustainable sourcing and accepted nutrition standards be integrated into food procurement as an intervention to promote sustainable and healthy food systems and diets [12–14]. The aim of this recommendation is to promote food safety and security, increase consumption of healthy and nutritious foods and improve the sustainability and efficiency of production, supply and distribution by establishing standards that guide how organisations procure, prepare, provide and sell foods [15]. To date, however, the strategic use of procurement has been limited, typically being employed as an administrative function, placing emphasis on competition, user value and cost [16, 17].

By implementing healthy and sustainable procurement policies, governments can use their purchasing power to drive market change and improve not only economic, but also social, environmental and health outcomes [14]. Additionally, it has been argued that government funds should not be spent on food that contributes to unhealthy diets and in turn the burden of disease and associated health costs later born by the government [18]. Provisioning and promoting healthy foods in public institutions via procurement can help mitigate adverse health outcomes, set a standard for private institutions and the community, ensure that contractual arrangements align with government policy, and show leadership in shifting social norms.

### Australian case studies

In Australia, significant work has previously been undertaken on the development of healthy food provisioning policies for schools and health facilities [15, 19, 20]. There has also been an increase in the development of sustainability policies and guidelines for general procurement [21–23]. Multiple government departments have been involved in the development of these policies but not always the implementation. While procurement is managed by one agency, often the Department of Finance or Treasury, healthy food provisioning policies are managed by others, such as, the Department of Health and the Department of Education with respect to healthy school canteen policies. In addition to this, agencies under the Department of Environment are involved in the consultation of sustainability policies. Multiple departments, each with varying policy agendas and incentives, are involved in different aspect of procurement which has the potential to cause policy incoherence. It is unclear how the relevant government agencies prioritise economic, health, environmental and social considerations.

It is also unclear whether and how existing nutrition and sustainability policies are embedded into food procurement contracts. For example, the Queensland Government’s *A better choice–healthy food and drink supply strategy* aims to increase healthier food and drink options to at least 80% of displayed foods and is supported by a mandatory healthy food supply directive [24, 25]. Yet, it is unclear if the requirements are incorporated into recent contracts, as the details are not publicly available. In South Australia, the *Sustainable Procurement Guideline* promotes strategies which avoid unnecessary consumption, promotes products and services that have lower environmental impact across the life cycle, and fosters local sustainability innovation [21]. However, there is no reference to sustainability as a requirement nor a consideration within the current health services food contract [26]. Policy and legislation provide the framework for how governments engage with current and potential suppliers from tender through to contract management, and what the key procurement priorities are. However, with contract negotiations being heavily guarded and often lacking transparency it is unclear how policy is implemented into contracts and what order priorities are given. Are all priorities equal? Are only some policies implemented?

This paper describes the protocol for undertaking a political economy analysis and will explore current policy implementation barriers, including why high-level policies aren’t embedded into contracts, mechanisms for achieving policy coherence and future opportunities for incorporating socio-economic, public health and environmental considerations into purchasing practices in Australia. To our knowledge, this is the first political economy analysis on food procurement in Australia.

## Methods

### Aims

The primary aim of this political economy analysis is to prospectively identify potential barriers and facilitators facing implementation and strategies for improving implementation of government policy. Using food procurement in Queensland and South Australia as case studies, the political economy analysis seeks to understand the governance and legal structures and the political economy drivers affecting implementation of existing healthy food provisioning policies and sustainable procurement guides into food procurement contracts.

Our research questions are:

- What are the structural and contextual factors of the food procurement process, and how do they influence the policy objectives?
- What are the bargaining processes within and external to government that influence the implementation of policies, the development of food procurement tenders and the awarding of contracts?
- Which stakeholders are included or excluded from the decision making and the bargaining processes, and what are the networks and connectors to the policy elite?
- What are the incentives and perceived benefits and trade-offs among government and industry stakeholders?

### Methodological and theoretical approaches informing our framework

Our research uses the political economy analysis methodology and draws on the World Bank’s *Problem-Driven Political Economy Analysis* and the Australian Department of Foreign Affairs’ *Political economy analysis guidance note*, which state that clear articulation of the policy problem and selection of the stage in the policy cycle are critical for ensuring practical relevance [27, 28]. In our case study, the policy problem is the lack of translation of high-level nutrition and sustainability policies into government food procurement contracts. As nutrition and sustainability policies already exist for food provisioning and procurement, we seek to understand the implementation readiness and feasibility of transferring policies into tender processes and contracts. If a theoretically good policy cannot be implemented in a local political setting, it is not a policy solution at all [14]. This is a novel approach to the area of food provisioning and procurement, where much of the work has focused on agenda setting or policy formation [29–32].

Political economy lacks a clear definition and political economy analysis frameworks vary because of this. For the purpose of this research we will be using the definition provided by Sparkes and colleagues, “political economy analysis is used to assess the power and position of key political actors, as a way to develop strategies to change the political feasibility of desired reforms” [33]. In building our political economy analysis framework we draw extensively on the political economy literature [9–11, 33] as well as policy implementation [34, 35] policy coherence [7] and political commitment literature [4, 5]. The politics of implementation focuses on the interests of actors, relationships between different stakeholders and their bargaining power–the elements that can lead to fracturing and an implementation deficit [35].

### The framework: Political economy analysis of food procurement

Our framework is intended to be used for prospective analysis of policy implementation barriers and enablers, focusing on the structural and contextual factors, bargaining processes, stakeholders, and incentives and ideas. In taking a prospective focus on implementation, we build on the prior work of Campos & Reich (2019) and Sparkes and colleagues (2019) on the politics of implementation, assessing the role and position of stakeholders and the power dynamics between them [33, 36]. These previous studies took a retrospective approach to health policy implementation.

**Fig 1:**
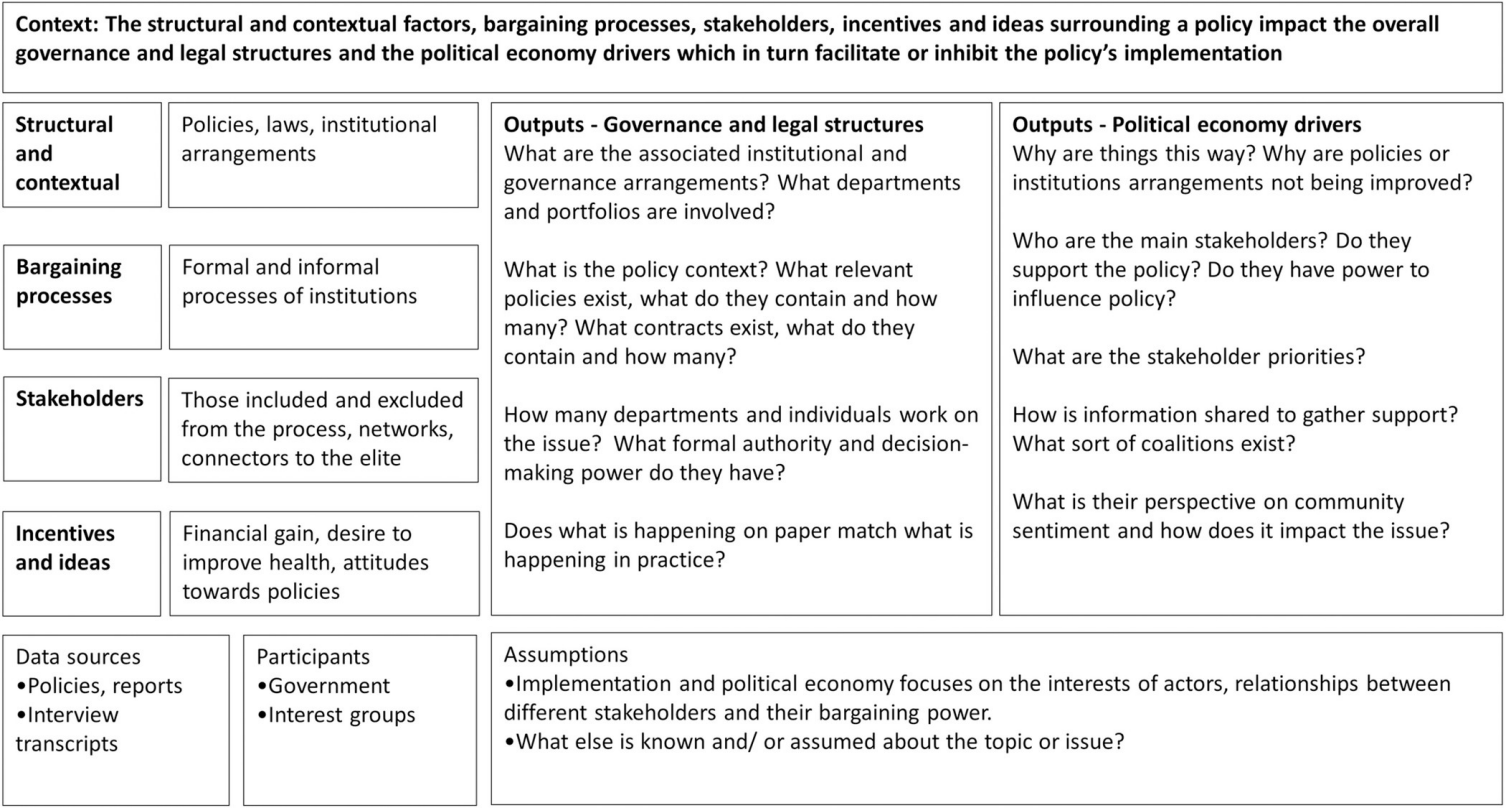
Political economy analysis framework for prospective policy implementation.

The political economy analysis framework for government food procurement (Fig 1) demonstrates the strategies used to identify the governance and legal structures–relevant policies, laws and departmental structures–and the political economy drivers–the key stakeholders, formal and informal processes, incentives and how the issue is framed. The framework has broad applicability and can be adapted for prospective analysis of policy implementation in a range of contexts.

With respect to our food procurement case study, the context is that implementation of existing nutrition and sustainability policies into food procurement contracts, may be inhibited due to certain political economy drivers in Queensland and South Australia. Queensland and South Australia were chosen as case studies as both governments have healthy provisioning and sustainable procurement policies, however, the contracts are managed and awarded differently, with South Australia having one contract with a panel of suppliers for all health services and Queensland having multiple contracts for each individual health service [37, 38]. The different structural arrangements may offer different insights into potential implementation barriers and enablers.

**Fig 2:**
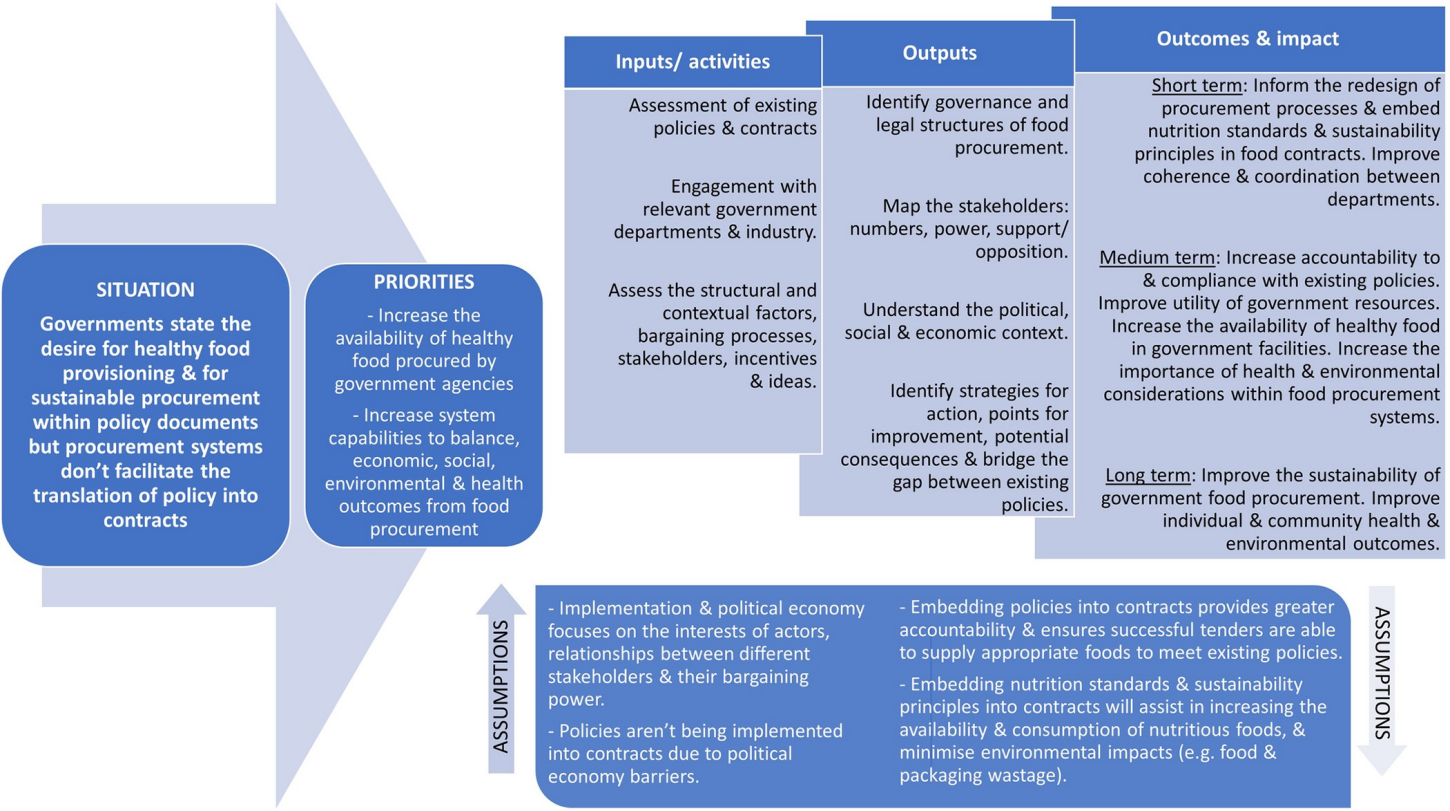
Logic model for embedding nutrition standards and sustainability principles into food procurement contracts.

The logic model (Fig 2) demonstrates how we anticipate the political economy analysis will support policy implementation and the outcomes and impacts over the short, medium and long term. In engaging with and identifying key stakeholders, assessing the structural and contextual factors, and understanding bargaining processes, incentives and ideas we are able to map the governance and legal structures and the political economy drivers for food procurement. This in turn allows us to collaboratively identify barriers and enablers and achieve impact by improving coherence between departments and elevating the priority of health and sustainability in procurement (short term); increasing the availability of healthy and sustainable foods (medium term) and improving health and environmental outcomes (long term).

The components of this context will be investigated through our framework using the following participants and data sources.

#### Participants

**Fig 3:**
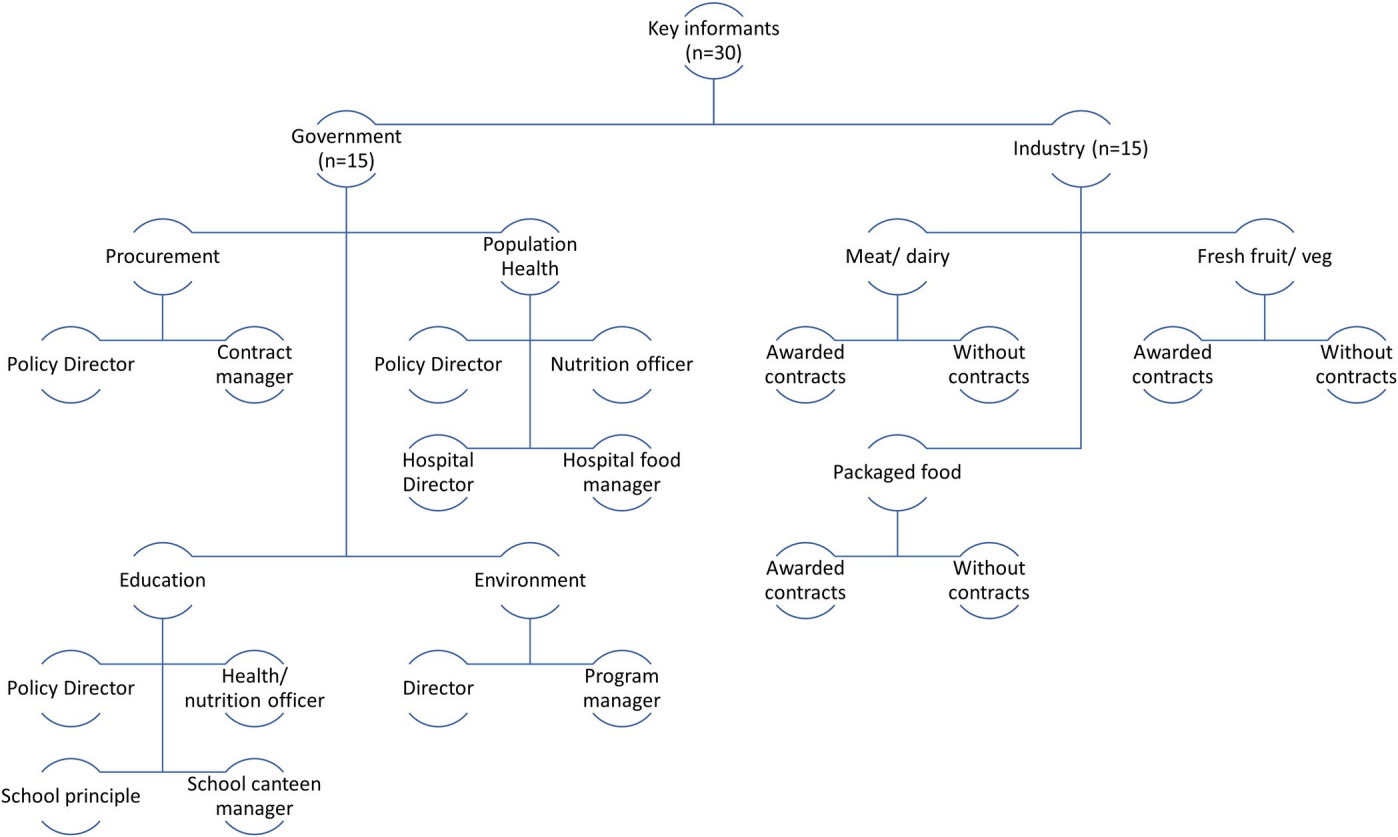
Sampling framework for government food procurement.

Government and industry stakeholders will be interviewed as these groups have the most intimate and relevant knowledge of the government food procurement process. Fig 3 outlines the sampling framework we plan to use and is a guide representing our ideal recruitment strategy. As different government departments have different policies and priorities, we plan to interview representatives from procurement, health, education and environment agencies or departments. Where possible, we plan to recruit individuals of varying managerial levels (for example, food managers, policy officers and department executives) to get a diverse understanding of institutional context and power dynamics.

Purposive sampling from The Australian Prevention Partnership Centre (TAPPC) network of policy partners will be used initially to recruit participants within government health departments with expertise in healthy food supply. TAPPC is a national collaboration of researchers, policy makers and practitioners, and is funded by and partnered with the National Health and Medical Research Council (NHMRC), the Australian Government Department of Health, ACT Health, Cancer Council Australia, NSW Ministry of Health, Wellbeing SA, Tasmanian Department of Health and VicHealth.

Participant recommendations will also be sought from a Technical Advisory Group of food policy experts, health economics and public health lawyers within a TAPPC public health law project [39]. This will be supported by snowball sampling to recruit participants within other government departments engaged in food procurement and industry stakeholders that frequently apply for or are invited to tender. If required, this will be supplemented by auditing organisational structures within government departments as well as current and past contracts for food suppliers.

Food contracts are generally awarded to multiple suppliers in different food categories. For this reason, we plan to recruit a range of suppliers via established contacts, including meat and dairy, fresh fruit and vegetables, beverages and packaged foods, to determine whether policies or processes affect different areas of the food industry more than others. To better understand bargaining processes and incentives and ideas we seek the perspectives of suppliers who have been awarded contracts, those who have been unsuccessful and if possible, those that wish to apply for contracts.

#### Data sources

Desktop analysis will be undertaken to identify existing policies and departmental structures as well as publicly available contracts from the past 10 years [40]. Government websites will be searched using key terms including “procurement policy”, “food procurement”, “sustainable procurement” and “healthy food provisioning”. SA Tenders and Contracts (www.tenders.sa.gov.au) and QTenders (https://qtenders.hpw.qld.gov.au/qtenders/) will be used to search for contracts. This will be supplemented by data from key informant interviews.

Key informant interviews will largely be used to identify the political economy drivers, mapping the stakeholders, bargaining processes, incentives and ideas. Topics covered in the interviews will include:

- Bargaining processes: How does the procurement process operate and how do different departments work together in these processes? What are the main aims of procurement and how is priority allocated? Is there negotiation on what is included or excluded from tenders and/or contacts?
- Stakeholders: Who are the main government departments involved in the creation and implementation of food procurement policies? Who are the main stakeholders applying for food contracts? What type of businesses would be most impacted by nutrition standards and/or sustainability principles?
- Incentives and ideas: What are the attitudes towards such provisions? How will it impact on supply, cost, quality, variety? What are the motivational factors for including or excluding provisions?

All interviews will be audio recorded, or online video recorded in the case that face to face interviews cannot be conducted, and transcribed. Recordings will be transcribed by a professional transcription service. The transcription service will sign a confidentiality agreement. Individual identifiers, direct (name, address) and indirect (gender, date of birth, profession) will be removed from the data during the transcription process and replaced with participant codes. Participants may request to review their transcripts.

### Analysis plan

A content analysis of the policy documents and contracts will be conducted to identify number and types of documents relating to food supply and procurement, and broader sustainable supply and procurement. Policy content will be assessed with respect to policy objectives and whether objectives and activities support or undermine nutrition and/ or sustainability.

Interview transcripts will be thematically analysed using an inductive and iterative coding approach moving from codes to categories to themes [41, 42]. Data coding and analysis will be performed throughout data collection. The development of the coding framework will be informed by the political economy analysis framework (Fig 1) and themes will be established under the framework headings: structures and context, bargaining processes, stakeholders, incentives and ideas. The analysis team will engage in frequent analytical conversations to identify and then explore themes. Themes will capture patterns from the data and the analysis team will engage in constant, iterative comparison of data within and between different stakeholder groups (e.g. procurement, health, meat suppliers, fruit suppliers, those with contracts, those without). It is anticipated that different stakeholder groups will offer different perspectives on the formal and informal processes, the networks and power dynamics, and the perceived benefits and trade-offs.

### Ethics

All participants will be provided information about the study and their written consent obtained. Ethics approval to conduct this research has been approved through the University of New South Wales, Australia (HC200340). In line with ethics requirements, data will be stored by The George Institute for Global Health on a UNSW supported storage platform (shared drive) only accessible by the research team. The data will be retained for a minimum of 7 years after completion of the project.

## Discussion

This framework is to be used for prospective, iterative use. We anticipate such a framework will be relevant for government officials, researchers or policy influencers seeking to identify barriers to implementation and strategies to overcome them. The case study presented is for food procurement, but the framework has broader applicability to public health policy. The data sources and participants within the framework can be adapted to the relevant context or problem. The results from this research will be disseminated to academic and non-academic audiences through peer-reviewed publications, conference presentations and formal and informal meetings with policymakers and practitioners.

### Outcomes and significance

Our study takes a novel approach to understanding the politics of implementation through a prospective Political Economy Analysis. In engaging with and understanding the structural and contextual factors, bargaining processes, stakeholders, and incentives and ideas, our findings will highlight barriers and enablers to implementation of healthy and sustainable food procurement policies. However, the outcomes will rely on the openness of stakeholders in providing information and will be limited if stakeholders choose to withhold or guard information regarding procurement processes and contract negotiations. To our knowledge this is the first political economy analysis of food procurement in Australia. Ultimately, the framework will achieve impact by improving coherence between departments and elevating the priority of health and sustainability in procurement (short term), increasing the availability of healthy and sustainable foods (medium term), and improving health and environmental outcomes (long term).

## References

[1] Swinburn BA, Kraak VI, Allender S, Atkins VJ, Baker PI, Bogard JR, et al. The global syndemic of obesity, undernutrition, and climate change: The Lancet Commission report. The Lancet. 2019;393.10173:791–846. 10.1016/S0140-6736(18)32822-8 30700377

[2] World Health Organization & Food and Agriculture Organization of the United Nations. Second international conference on nutrition: report of the joint FAO/ WHO Secretariat on the conference, December 2014. FAO and WHO; 2015.

[3] Post LA, Raile ANW, Raile ED. Defining Political Will. Politics & Policy. 2010;38(4):653–76.

[4] Baker P, Hawkes C, Wingrove K, Demaio AR, Parkhurst J, Thow AM, et al. What drives political commitment for nutrition? A review and framework synthesis to inform the United Nations Decade of Action on Nutrition. BMJ Global Health. 2018;3(1):e000485. doi: 10.1136/bmjgh-2017-000485 29527338 PMC5841521

[5] Gillespie S, Haddad L, Mannar V, Menon P, Nisbett N. The politics of reducing malnutrition: building commitment and accelerating progress. Lancet. 2013;382(9891):552–69. doi: 10.1016/S0140-6736(13)60842-9 23746781

[6] Baker P, Kay A, Walls H. Trade and investment liberalization and Asia’s noncommunicable disease epidemic: a synthesis of data and existing literature. Globalization and Health. 2014;10(1):66. doi: 10.1186/s12992-014-0066-8 25213212 PMC4180923

[7] Thow AM, Greenberg S, Hara M, Friel S, duToit A, Sanders D. Improving policy coherence for food security and nutrition in South Africa: a qualitative policy analysis. Food Security. 2018;10(4):1105–30.

[8] OECD. Better policies for sustainable development 2016: A new framework for policy coherence. Paris: Organization for Economic Cooperation and Development (OECD) Publishing; 2016.

[9] Pinstrup-Andersen P. The political economy of food and nutrition policies. Baltimore, MD: Published for the International Food Policy Research Institute (IFPRI) by Johns Hopkins University Press; 1993.

[10] Reich MRB, Yarlini. Political Economy Analysis for Food and Nutrition Security. Health, Nutrition and Population (HNP) discussion paper. Washington DC: World Bank; 2012.

[11] Reich MR, Balarajan Y. Political economy analysis for nutrition policy. Lancet Glob Health. 2014;2(12):e681–2. doi: 10.1016/S2214-109X(14)70350-X 25433616

[12] Environment UN. 2017 Global review of sustainable public procurement. 2017.

[13] World Health Organization. Action framework for developing and implementing public food procurement and service policies for a healthy diet. Geneva: World Health Organization (WHO); 2021.

[14] Food and Agriculture Organization of the United Nations. Strengthening sector policies for better food security and nutrition results: political economy analysis. Food and Agriculture Organization of the United Nations (FAO); 2017.

[15] Rosewarne E, Hoek AC, Sacks G, Wolfenden L, Wu J, Reimers J, et al. A comprehensive overview and qualitative analysis of government-led nutrition policies in Australian institutions. BMC Public Health. 2020;20(1):1038. doi: 10.1186/s12889-020-09160-z 32605547 PMC7325668

[16] OECD. Progress Made in Implementing the OECD Recommendation onEnhancing Integrity in Public Procurement. Report to Council. Organisation for Economic Co-operation and Development (OECD); 2012.

[17] Morgan K, Sonnino R. The school food revolution: public food and the challenge of sustainable development: Routledge; 2013.

[18] WHO urges governments to promote healthy food in public facilities [press release]. Geneva: World Health Organization 2021.

[19] ACT Government. ACT public sector healthy food and drink choices policy. Policy Number: WHS-01-20162016.

[20] Department of Health Victoria. Healthy choices: policy directive for Victorian public health services. State of Victoria; 2021.

[21] Government of South Australia State Procurement Board. Sustainable Procurement Guideline. 2017.

[22] Australian Government. Sustainable Procurement Guide: A practical guide for Commonwealth entities. Canberra: Commonwealth of Australia; 2020.

[23] The Office of the Chief Advisor Procurement. Integrating sustainability into the procurement process. Brisbane: The State of Queensland (Department of Housing and Public Works); 2018.

[24] Queensland Government. A better choice—healthy food and drink supply strategy. Brisbane: Queensland Health; 2019.

[25] Queensland Health. Health Service Directive—Healthier Food and Drinks at Healthcare Facilities. QH-HSD-049:2019. Brisbane: Queensland Government; 2020.

[26] General Food and Beverage Panel Contract ID SAH2012–191. SA Health and Barker Boy Processing. Execution date 27 November 2014; completion date 30 September 2021. Accessed 2 September 2021 https://www.tenders.sa.gov.au/contract/view?id=186313.

[27] Fritz V, Levy B, Ort R. Problem-Driven Political Economy Analysis: The World Bank’s Experience. Washington DC: World Bank; 2014. Contract No.: License: CC BY 3.0 IGO.

[28] Department of Foreign Affairs. Political Economy Analysis Guidance Note Canberra: Australian Government; 2016.

[29] Raine KD, Atkey K, Olstad DL, Ferdinands AR, Beaulieu D, Buhler S, et al. Healthy food procurement and nutrition standards in public facilities: evidence synthesis and consensus policy recommendations. Health promotion and chronic disease prevention in Canada: research, policy and practice. 2018;38(1):6–17. 29323862 10.24095/hpcdp.38.1.03 PMC5809107

[30] Niebylski ML, Lu T, Campbell NRC, Arcand J, Schermel A, Hua D, et al. Healthy food procurement policies and their impact. International journal of environmental research and public health. 2014;11(3):2608–27. doi: 10.3390/ijerph110302608 24595213 PMC3986994

[31] Waterman C, Feldman M, Kraus-Polk J. Food Procurement and Infrastructure. 2021.

[32] Alberdi G, Begiristain-Zubillaga M. Identifying a Sustainable Food Procurement Strategy in Healthcare Systems: A Scoping Review. Sustainability 2021, 13, 2398. s Note: MDPI stays neutral with regard to jurisdictional claims in published …; 2021.

[33] Sparkes SP, Bump JB, Özçelik EA, Kutzin J, Reich MR. Political Economy Analysis for Health Financing Reform. Health Systems & Reform. 2019;5(3):183–94. doi: 10.1080/23288604.2019.1633874 31369319

[34] Pressman J, Wildavsky A. Implementation: how great expectations in Washington are dashed in Oakland, or, why it’s amazing that federal programs work at all, this being a saga of the economic development administration as told by two sympathetic observers who seek to build moral. Berkeley: University of California Press; 1973.

[35] Hill M, Hupe P. The multi-layer problem in implementation research. Public Management Review. 2003;5(4):471–90.

[36] Campos PA, Reich MR. Political Analysis for Health Policy Implementation. Health Systems & Reform. 2019;5(3):224–35. doi: 10.1080/23288604.2019.1625251 31390295

[37] Government of South Australia. SA Tenders & Contracts 2021. Available from: https://contracts.sa.gov.au/welcome.

[38] Queensland Government. QTenders 2021. Available from: qtenders.hpw.qld.gov.au.

[39] The Australian Prevention Partnership Centre. Public health law: making it work for the prevention of chronic disease: Sax Institute; 2021 [Available from: https://preventioncentre.org.au/our-work/research-projects/public-health-law-making-it-work-for-the-prevention-of-chronic-disease/.

[40] Sabatier P. Theories of the Policy Process. 1 ed: Routledge; 2007.

[41] Braun V, Clarke V. What can "thematic analysis" offer health and wellbeing researchers? Int J Qual Stud Health Well-being. 2014;9:26152. doi: 10.3402/qhw.v9.26152 25326092 PMC4201665

[42] Braun V, Clarke V. Using thematic analysis in psychology. Qualitative Research in Psychology. 2006;3(2):77–101.

